# Reducing or reproducing inequalities in health? An intersectional policy analysis of how health inequalities are represented in a Swedish bill on alcohol, drugs, tobacco and gambling

**DOI:** 10.1186/s12889-022-13538-6

**Published:** 2022-07-07

**Authors:** Nadja Fagrell Trygg, Per E. Gustafsson, Anna-Karin Hurtig, Anna Månsdotter

**Affiliations:** Department of Epidemiology and Global Health, Umeå University, 901 87 Umeå, Sweden

**Keywords:** Intersectionality, Post-structural policy analysis, Alcohol, Drugs, Tobacco, Gambling, Health inequalities, Health policy

## Abstract

**Background:**

According to post-structural policy analyses, policies and interventions aiming at reducing social inequalities have been found to be part in producing and reifying such inequalities themselves. Given the central role of health inequalities on the public health policy agenda globally it seems important to examine the way policy on health inequalities may potentially counteract the goal of health equity*.* The aim of this intersectional policy analysis, was to critically analyze the representation of health inequalities in a government bill proposing a national strategy on alcohol, drugs, tobacco and gambling, to examine its performative power, and to outline alternative representations.

**Method:**

A post-structural approach to policy analysis was combined with an intersectional framework. The material was analyzed through an interrogating process guided by the six questions of the “What’s the problem represented to be?” (WPR) approach. Thus, the underlying assumptions of the problem representation, its potential implications and historical background were explored. In a final step of the analysis we examined our own problem representations.

**Results:**

The recommendations found in the gender and equity perspective of the bill represented the problem of health inequalities as a lack of knowledge, with an emphasis on quantitative knowledge about differences in health between population groups. Three underlying assumptions supporting this representation were found: quantification and objectivity, inequalities as unidimensional, and categorization and labelling. The analysis showed how the bill, by opting into these partly overlapping assumptions, is part of enacting a discourse on health inequalities that directs attention to specific subjects (e.g., vulnerable) with special needs (e.g., health care), in certain places (e.g., disadvantaged neighborhoods). It also showed how underlying processes of marginalization are largely neglected in the bill due to its focus on describing differences rather than solutions. Finally, we showed how different intersectional approaches could be used to complement and challenge this, potentially counteractive, problem representation.

**Conclusions:**

The problem representation of health inequalities and its underlying assumptions may have counteractive effects on health equity, and even though some of its strengths are raised, it seems to be profoundly entangled with a system resisting the kind of change that the bill itself advocates for. If carefully used, intersectionality has the potential to support a more comprehensive and inclusive equality-promoting public health policy and practice.

## Background

Tackling health inequalities has been on the public health policy agenda globally, at least since the WHO launched the global Policy on “Health for all by year 2000” [1]. In this context, harmful use and misuse of alcohol, drugs, tobacco and gambling is a major contributor to injuries, sickness, mortality, and inequalities in health worldwide. It has been an explicit target area of Swedish public health policy since 2002 [2], which more recently also has incoroporated a stronger emphasis on health equity. In this study, we analyze how the concept of health inequality is represented in a 2021 Swedish government bill for a renewed strategy on alcohol, drugs, tobacco and gambling, which is one of the most comprehensive strategic areas of national public health in Sweden [3]. We combine a post-structural approach to policy analysis [4, 5] with an intersectional framework [6] to interrogate the performative power of the policy representation and to suggest transformative alternatives.

### Post-structural policy analysis

Post-structural policy analysis center around how policies and other governing technologies themselves are part in constituting the problem that they are intended to solve [5]. This perspective draws on ideas like performativity and ontological politics [7] and has received increased attention lately in policy research and analysis (see e.g.: [8–12]). Such studies have examined the role of census registers [13], statistical data [14], evidence-based policy [15, 16], international migration policy [17] asylum policy [18], and representations of for example, gender [19–21], sport participation [22], alcohol [23], drugs [8, 24, 25], women’s alcohol consumption [26] drug use among lesbian, gay, bisexual, transgender, intersex, and questioning [27] and vulnerability [28, 29]. Through these studies the role of policy and research in shaping ways of thinking, subjects and their lives are illustrated. For example, Rowse [13] showed how the national census of Australia as a governing technology played an important role in constituting the “the national population” and other “sub-populations” such as the binary categories “Indigenous” and “non-Indigenous”. Thus, the census had implications beyond being “just a register”; it was a necessary actor in making these subjects thinkable and governable. Another example is a study by Martin and Aston [20] who analyzed research on women drug users and found them to be represented as a “special population” with “special needs”. This had implications on the understanding of the health situation of the group and how support could be effectively provided. Brown and Wincup [29] came to similar conclusions after analyzing current English drug policy and how the concept of “vulnerability” was represented. They argue that the present problematization of vulnerability hid the complexity behind drug-related health inequalities by downplaying the role of material and social inequalities. Therefore, the authors suggested alternative representations with potential to produce deeper understandings and solutions to drug-related health inequalities focusing on the diverse and multiple processes of marginalization involved.

### The WPR-approach

The specific post-structural approach guiding the policy analysis of this paper is called the What’s the Problem Represented to be, or in short: the WPR-approach. The analytical tool consists of a set of questions and a final step presented in Table 1. It is used to expose the deeper, underlying and implicit problem, beyond the explicit issue that the policy intends to solve [4]. For example, a policy aiming at reducing harmful alcohol drinking by putting restrictions on the supply (availability) of alcohol, represents the problem as the availability of alcohol [23]. Thus, such policy may constitute subjects as lacking self-regulation and risks representing the cause of the problem as grounded in individual weakness rather than the conditions surrounding the individual [23]. Through this kind of analysis the WPR-approach strives to develop interventions, or modes of control, that follows the ethical principle of Foucault, to produce “as little domination as possible” [30].

**Table 1:** Overview of the questions and final step covered by the WPR-approach. Adapted from: Bacchi and Goodwin [5]

WPR Chart: What’s the Problem Represented to be? (WPR approach to policy analysis)
Question 1: What’s the problem (e.g. of “gender inequality”, “drug use/abuse”, “economic development”, “global warming”, “childhood obesity”, “irregular migration”, etc.) represented to be in a specific policy or policies?
Question 2: What deep-seated presuppositions or assumptions (conceptual logics) underlie this representation of the “problem” (problem representation)?
Question 3: How has this representation of the “problem” come about?
Question 4: What is left unproblematic in this problem representation? Where are the silences? Can the “problem” be conceptualized differently?
Question 5: What effects (discursive, subjectification, lived) are produced by this representation of the “problem”?
Question 6: How and where has this representation of the “problem” been produced, disseminated and defended? How has it been and/or how can it be disrupted and replaced?
Step 7: Apply this list of questions to your own problem representations

Furthermore, the WPR-approach is closely related to the broader study of governmentality also formulated by Foucault [31] and further developed by others [32, 33]. The study of governmentality directs attention to the underlying rationalities and technologies of power, including biopower or biopolitics. Biopolitics refers to the diverse technologies (i.e., modes) controlling the health and bodies of populations [32] such as policies restricting the availablity of alcohol. The WPR-approach links to the study of governemntality by examining discursive, subjectification and lived effects of policy and by raising the historical dependency of the policys’ underlying assumptions. Discursive effects include limits of what can be thought and said, subjectification effects include the production of specific “subjects” and, lived effects are the result of the two previous effects on people lives, behaviors or material conditions [5]. Tracking the history of an underlying assumption is thought to have a destabilizing effect on discourse, subjects and objects which is why the analysis also involves tracing historical elements of the underlying assumptions (similar to question 3 and 6, Table 1). The final step of the WPR-approach is an examination of one’s own problem representation (step 7, Table 1).

### Swedish public health policy and strategy to reduce harm from alcohol, drugs, tobacco and gambling

#### A target area in public health policy

Alcohol, drugs, tobacco and gambling has been an explicit target area in the Swedish public health policy since 2002 [2]. At that time, the national public health policy included 11 target areas of monitoring and intervention of which the last concerned alcohol, drugs, tobacco and gambling, aiming for a reduction in tobacco and alcohol use, a society free from drugs, and fewer negative consequences from gambling. The reason behind the specific attention to these health behaviors were their contribution to injuries, sickness, and mortality, the human and monetary costs on family and society, and the gender and socioeconomic inequalities linked to use and harm. In the renewed national public health policy from 2018 [34], alcohol, drugs, tobacco and gambling were however grouped with other health related behaviors.

Initially, specific plans were developed and implemented according to health behavior as a way to achieve the targets set out by the public health policy. As the national plans on alcohol and drugs were successfully implemented, the national audit office recommended to combine the plans and to include tobacco prevention. These combined and expanded plans paved the way for the first government strategy on alcohol, drugs and tobacco which was adopted by the Swedish parliament 2010 [35]. As part of the strategy an idea of gender mainstreaming was formulated together with an idea of the importance of tailoring interventions for individual needs and difficulties such as immigration or disability related difficulties [35]. However, a concept of health inequality was not explicitly described, and the term inequality or equality, apart from gender inequality, was not used.

### A strategy with an equity perspective

In 2015 the strategy on alcohol, drugs and tobacco was updated and the new overarching aim of the national public health policy – to create good societal conditions for equitable health in the entire population and to “close the avoidable health gaps in a generation” [34] was considered central [36]. In the renewed strategy, gender mainstreaming was paired with an idea of equity mainstreaming [36]. The particular perspective to be mainstreamed into the strategy was also described.

In the evaluation of the strategy period 2006–2020, the Public Health Agency of Sweden (that had been appointed the national responsibility to support implementation through co-ordination and monitoring as well as knowledge development and dissemination) concluded that there was no indication of reduced inequalities in relevant health outcomes and that gender and equity mainstreaming had not been fully implemented, particularly on the national level [37].

In 2021 further amendments to the strategy were proposed in a government bill [3] in which nicotine products without tobacco, and gambling, were added. The inclusion of these substances and behaviors in the same strategy was considered a strength since it allowed for concerted action on common social determinants, risk factors, and areas of prevention. The recommendation to mainstream a gender and equity perspective remained but, despite the results from the evaluation regarding insufficient implementation no further guidance on this was provided.

### Overview of the 2021 government bill

The strategy proposed in the government bill [3] is structured around seven target areas which make up the bulk of the content: Reduce access; Protect children and youth; Reduce use and delay debut in children and youth; Reduce harmful use, misuse and addiction; Accessible person-centred quality health care for persons with addiction and abuse; Reduce injuries and mortality; and Contribute to international and European collaboration. Each target area has a subheading “prioritized focus areas,” in total 29 areas, in which actions proposed by the government are presented. Furthermore, an organisation for the implementation of the strategy is laid out. Actors on all three governing levels (national, regional and local) are appointed responsibility for the implementation of the strategy. Central to this study is that the entirety of the strategy is supposed to rest on eight value-perspectives related to: Risk and target groups; Collaboration; Gender equity and equity; Protection of children and youth; Convention on the right of the child; Relatives and close relatives; Agenda 2030 on sustainable development; and Impact on environment.

In the government bill [3], the need for targeting health inequalities is motivated by two main arguments. One is related to health as a human right quoting the formulation in the Convention of economic, social and cultural human rights from 1966 “the right of everyone to the enjoyment of the highest attainable standard of physical and mental health”. The other is related to the national public health policy aim to “close the avoidable health gap in a generation” [34]. These arguments reflect an underlying logic representing health inequalities as a sign of failure in achieving these two ambitions, thus motivating a policy on this problem (of health inequalities).

### Intersectional policy analysis

In order to advance the equity perspective of health policy and bring about a conceptual shift in how health inequalities are approached, intersectional policy analysis has been proposed [10]. Such analysis is suggested to go beyond stigmatized understandings of “vulnerable groups” or “special populations” with “special needs” and unidimensional approaches to monitoring and health impact assessment. The concept of intersectionality is concerned with relationships of mutually constituting structural disadvantage. In its original application by Crenshaw in 1989, it was used to show how gender and racial structures of power worked together in the marginalization of women of color [38]. Intersectional policy analysis aims at transformative knowledge that challenges and disrupts present mechanisms of oppression and marginalization by for example, pointing out such mechanisms [10].

Overall, intersectionality has been conceived and approached in many ways, for example as a theoretical framework [39], a hypothesis [40], a research paradigm [40], thinking technology [41], analytic sensibility [42], and a social movement [43] all emphasizing the relationship and entanglement of multiple dimensions of inequality and oppression. McCall [6], has provided a heuristic to facilitate a general understanding of different intersectional approaches: the intra-categorical, the anti-categorical and the inter-categorical. According to McCall, different approaches have developed in conversation and debate with each other as well as other feminist theories of science. In summary, the intra-categorical approach is grounded in critique raised by feminists of color, stressing the need for voicing the experiences at neglected intersecting points, such as the experiences of black women. The anti-categorical approach is grounded in post-structural and postmodern ideas, applied mainly through methods of deconstruction and with the aim to reveal the social constructedness of analytical categories and thus liberating individuals tied to the norms and stereotypes associated with a particular category. The inter-categorical approach focuses on the relationship between intersectional groups (e.g., white men and black women) and is approached quantitatively, preferably with large data sets which is probably why it has been a useful starting point for epidemiological intersectionality research [39, 44].

Even though intersectionality has been raised as a promising framework to advance public health policy and health inequality research [10, 44], its use has been found to be fragmented and not always clearly linked to theoretical tenets [39, 45]. Lapalme, Haines-Saah and Frohlich [46] perceives a limitation in that current public health research using intersectionality tends to focus on the experiences of marginalized groups and not so much on the underlying mechanisms reproducing inequalities and the power structures shaping the experiences of marginalization. Similarly, Bauer and Scheim discuss and propose methods within intersectional methodology in order to push its concern beyond what they call descriptive intersectionality and towards analytical intersectionality focusing on underlying causal processes [47, 48].

When it comes to earlier applications of intersectionality to public health policy analysis none have specifically focused on the concept of health inequalities and only a few have focused on topics related to alcohol, drugs, tobacco or gambling. Nevertheless, Hunting [49] examined a Canadian policy addressing Fetal Alcohol Spectrum Disorder (FASD) explicitly using an intersectional perspective. The analysis showed how underlying assumptions of gender, risk and culture reflected in the problem representation of FASD eventually reaffirmed a discourse and construct of FASD as a problem of individual women who make poor decisions. Recognizing such power of policy and its unintended, potentially counteractive effects on health equity, it seems important to critically analyze public health policies, such as the government bill proposing a strategy on alcohol, drugs, tobacco and gambling.

### Aim

The aim of this intersectional policy analysis, was to critically analyze the representation of health inequalities in a government bill proposing a national strategy on alcohol, drugs, tobacco and gambling, to examine its performative power, and to outline alternative representations.

In relation to this overall aim the following research questions were formulated:

1. How are health inequalities represented?
2. What assumptions underlie this representation and what are their implications?
3. How does intersectionality contribute with alternative representations?

## Material and Method

The material used for this analysis is the section of the government bill [3] “A gender and equity perspective should permeate the work”, describing the gender and equity perspective that the rest of the strategy should consider (17-20 pp). This perspective can be described as an empirical background informed through references to research and population statistics with a couple of recommendations and does not articulate any theoretical principles. By applying the gender and equity perspective to the rest of the strategy proposed in the bill, inequalities in health (related to alcohol, drugs, tobacco and gambling) are supposed to be reduced. Thus, it can be seen as “a policy within a policy”, that is, a policy on how to approach the rest of the targets and prioritized focus areas of the strategy. However, no direct guidance on how this “policy” could be implemented in organisations, projects or practices is given.

### Analysis

The material was analyzed by examining the text based on the questions of the WPR-approach (Table 1; [5]) together with intersectionality as outlined by McCall [6] as a theoretical framework.

The analysis starts by examining the problem representation of inequalities in the government bill (question 1, Table 1). However, we did not focus on the primary problem representation in terms of a lacking gender and equity perspective in the implementation of the strategy. Rather, our analysis started by examining how the gender and equity perspective of the bill was represented. We continued the analysis by asking which assumptions supported this particular gender and equity perspective (similar to question 2, Table 1). These underlying assumptions were identified through the intersectional framework [6] with which we also examined potential implications of the assumptions in terms of discursive, subjectification, and lived effects (similar to question 5, Table 1). This included the identification of exclusions (similar to question 4, Table 1) that is, voices and discourses left out from the gender and equity perspective of the government bill. Furthermore, we traced historical elements of these underlying assumptions (question 3, Table 1) and, with respect to each underlying assumption we also explored how these could be disrupted or replaced (similar to question 6, Table 1) by intersectional alternatives.

The material was analyzed as a coherent hole, that is, it was never broken up into smaller units or codes to be categorized. This was done to make a holistic interpretation of the text rather than a literal interpretation which we believe would have been misleading. The procedure of asking questions and looking for answers in the material was repeated several times in order to exhaust the material and minimize the risk of missing important information or misinterpretation. The final reflection was done when answers to the six questions had been formulated.

By combining the WPR-questions with intersectionality, the policy representation of health inequalities was not examined unconditionally but through the three approaches to intersectionality as outlined by McCall [6]: the intra-categorical, anti-categorical, and inter-categorical approach. This restriction to the analysis is discussed in a final reflection of the article. However, by combining the WPR-questions with intersectionality we intended to answer the call by Lapalme, Haines-Saah and Frohlich [46] to put power relations at the centre of intersectionality research.

### Reporting of results

We start by outlining how health inequalities are represented in the government bill, corresponding to research question 1. We continue by describing three underlying assumptions of the representation identified in the analysis, corresponding to research question 2. With respect to each underlying assumption, we also illustrate intersectional alternatives, corresponding to research question 3.

## Results

### Representation of health inequalities

The analysis of the problem representation showed that producing and using knowledge about differences in risk and health outcomes was a central solution to health inequalities. Analogously, the lack of knowledge about such differences are represented as a problem. The differences are formulated as quantitative phenomenon in terms of a gap or gradient in health outcomes and behaviors between population groups, thus emphasizing a certain type of knowledge (i.e., based on quantitative data). The main recommendations expressed in the gender and equity perspective of the government bill were: 1) knowledge about how risk factors affect population groups differently should be obtained and consider when developing preventive measures; for example, that women develop alcohol related harms on a lower level of alcohol consumption, and 2) that equity-oriented health promotion and disease prevention should focus on highlighting differences between men and women and between other sociodemographic groups.

The groups are mostly defined by a single dimension of inequality such as gender, socioeconomic position or sexual orientation, but sometimes two dimensions are cross-classified (combined). For example, the groups “older women,” and “women with disabilities” are described with respect to smoking prevalence, and with respect to harmful use of alcohol the socioeconomic gradient is described to be particularly evident among men. Hence, the differences in health are represented as a relation between certain types of groups. Furthermore, some groups are described as “frail,” or “vulnerable,” and some groups as “left behind,” or as “running particularly great risk of being affected by disease”. Lesbian, gay, bi- and transsexual people are for example “vulnerable,” people with recent migration experience and children “frail,” and women with addiction “particularly vulnerable”.

A central statement made in the government bill is that inequalities in health are caused by unequal and inequitable life opportunities and life conditions (as opposed to individual behaviour). The interplay of multiple life conditions in their effect on health behaviour is also raised as important, but neither motivated nor exemplified. Even though health inequalities linked to alcohol, drugs, tobacco and gambling are understood as caused by unequal life conditions, the bill is unclear about which life conditions and how they cause such inequalities in health.

The gender and equity perspective, outlined above, supports three, partly overlapping underlying assumptions: quantification and objective knowledge, inequalities as unidimensional and, categorization and labelling. We continue by tracing their historical background and by elaborating on the assumptions using an intersectional lens [6], examining potential implications in terms of discursive, subjectification and lived effects, including potential exclusions. Finally, we discuss how the different intersectional approaches could potentially challenge these assumptions and transform the gender and equity perspective of the government bill.

### Underlying assumptions and implications

#### Underlying assumption 1: Quantification and objective knowledge

Even though not explicitly stated, the government bill on alcohol, drugs, tobacco and gambling reflects a quantitative epistemological assumption in which numbers play a dominant and central role in representing the problem. This is not unexpected given the central role of epidemiology in public health sciences, and the history of epidemiology goes hand in hand with the science of statistics that emerged in the seventieth century [50]. In Sweden, the statistical record of the population is often described as particularly old and well preserved. It started with the church law on bookkeeping in 1686 paving the way for the precursor of todays’ Statistics Sweden which introduced population statistics in 1749 [51]. The statistics included sociodemographic information such as age, civil status, and family size as well as information about health behaviors. This system of registration and quantification of population groups have played an important role in securing citizen rights and benefits such as pensions, child support and health care, but also in the discrimination of population groups that were not considered to be Swedish, such as the Sami and Romani populations, who did not have the same entitlements. Because of fear of risks for exploitation, discrimination and prosecution of minority populations, registration of for example, ethnicity is today considered particularly sensitive personal information that is not permitted to be recorded in routine population registers. However, the lack of knowledge about for example, health in ethnic minority groups is nevertheless raised in the political debate as problematic.

The use of numbers to describe health inequalities may reflect an underlying assumption that numbers are objective, as in value-free [52]. The legacy of such value-free standard of science has been deeply challenged by feminist movements pointing out gender biases related to both scientific results and the scientific society overall [53]. Haraway [54] called it “the god trick;” the rhetoric that guises the specific power position contained in a policy with “objective,” and “neutral” numbers and disqualifying all other positions and knowledges as “subjective,” and “biased”. This way of thinking about knowledge as true or false, objective or subjective is a discursive effect that constitutes the subjectification of for example, “experts,” and “lay public” and maintains the voice and standpoint of the marginalized excluded and othered [54]. Similarly, it reflects a way of thinking about the dimensions of inequality such as gender, race and ethnicity as “true” entities readily quantifiable and measurable.

### Intersectional alternatives

The intra-categorical approach adopts the idea of standpoints and situated knowledges calling for dialogue and diversification in knowledge production [55]. In contrast to an objective and value free standpoint, the intra-categorical approach suggests that stand points particularly marked by experiences of multiple marginalization, enables a different understanding of how underlying systems of oppression operate [56]. Such understanding is recognized as equally valuable and “true” as the dominant standpoint. The typical study then focuses a single social group but could also focus a social setting or an ideological construct [6]. The intra-categorical approach challenges the assumption of objectivity reflected in the government bill and could be used to proliferate the gender and equity perspective by including voices from different standpoints.

The anti-categorical approach lies at the end of the continuum of critique of the scientific method, its claims to objectivity and truth [6]. It goes beyond the intra-categorical critique by fully rejecting the possibility of giving oppressed groups (or any group) a common voice at all. The anti-categorical method of deconstruction, is seen as a means of change, a way to alter social practices by liberating individuals from social norms, rather than an alternative way of obtaining objective knowledge. Dimensions of inequality are seen as unstable characteristics without an essence to objectively measure. Thus, the anti-categorical approach clearly challenges the assumption of fixed categories readily quantifiable. The idea of socially constructed categories eschews determinism and has the potential to contribute with knowledge-making practices that enacts empowered individuals with agency.

The inter-categorical approach provides a middle way. Even though dimensions of inequalities are seen as unstable they can still be measured and used provisionally and to define groups. But, since the analysis adds complexity by showing how inequalities manifest in different ways in different context this approach does not assume a positivist stance according to McCall [6]. Thus, the inter-categorical approach challenges the assumption of objectivity by highlighting the shifting meaning of categories and inequalities however, without completely rejecting the fundamental element of quantification.

### Underlying assumption 2: Inequalities as unidimensional

A second underlying assumption, reflected in the government bill on alcohol, drugs, tobacco, and gambling, is that of health inequalities as unidimensional. This is also mostly how inequalities historically have been approached, either as a class movement or a feminist movement or an antiracism movement [40] which is also reflected in the way policy and academia has approached health inequalities; either focusing on gender [57], or socioeconomic position [58], or ethnicity [59]. The discursive effect is an either-or way of thinking, assuming solidarity and uniform experiences among subjects belonging to the same category. This has been associated with identity politics and an “oppressions Olympics” in which oppressed and marginalized groups compete over resources rather than collaborate [40, 55]. And, as original intersectional critique brings up, the lived effect is an interlocking prison for example black women who will not privilege a single aspect of their identity in favour of either the white-dominated feminist movement or the man-dominated antiracist movement [60].

Another intersectional critique of the unidimensional approach to health inequalities highlights the risk of obscuring inequalities by comparing averages of population groups and blaming the victim by treating the dimensions of inequalities as individual risk factors [61]. Furthermore, the underlying causes of ill-health are likely to be very different in unidimensional population groups, such as “women” in which for example, an immigrant working class woman and a non-immigrant upper-class woman are clustered together but may have very different health needs [62]. Even though not explicitly mentioned in the analysed bill, there is an implicit call for an intersectional approach to better understand how life conditions taken together affect health. And, even though often unidimentionally represented, binary gender categories are sometimes nuanced by a cross-classification with age and socioeconomic position.

The stratification of gender and age can be traced historically to the epidemiological tradition of keeping morbidity and mortality registers stratified according to gender and age (see e.g., [63]). This was however primarily done to reflect biological and demographic differences rather than gender inequalities or inequities. Since the reason behind stratification is not always explicitly stated or theoretically informed, gender and age as dimensions of inequalities or as biologically caused differences, could be confused. For example, it is stated in the bill that it is important to consider that women develop alcohol related harms on a lower level of alcohol consumption and that health promotion and disease prevention should focus on highlighting differences between men and women, and other sociodemographic groups. Following such guidance, without elaborating on explanations to such differences, could further increase the risk of confusing dimensions of inequalities with their causes, essentialising population categories, and blaming them rather than pointing towards actionable solutions.

### Intersectional alternatives

The intra-categorical approach, can provide an alternative to the unidimensional representation. Traditionally this has been done using qualitative methods exploring intersectional locations or experiences from an intersectional standpoint [6]. The approach has the potential to go beyond the description of mere differences in health and to examine underlying processes and structural drivers of health inequalities such as racism and other forms of discrimination. There are also quantitative interpretations of the intra-categorical approach based on stratified analyses within a selected sample [39]. This produces a limited number of intersections, making the statistical analysis feasible in most cases. The approach could however also be criticised for fuelling an “oppression Olympics” in the search for the “most marginalized group” [55]. In summary, the intra-categorical approach challenges the assumption of health inequalities as unidimensional by showing how intersectional groups are marginalized. It also has the potential to deepen the gender equality and equity perspective of the government bill by providing knowledge about underlying processes of inequality.

The anti-categorical approach provides a fundamentally different way of thinking about inequalities than both the unidimensional and the intra-categorical way of thinking. It has traditionally been used to raise awareness about the constructedness of for example, “womanhood,” or “working classness,” and suggests that these constructs are fundamental to discriminating practices. An important step towards equality is therefore to abandon such categorization and essentialisation all together. Thus, the anti-categorical approach does not just challenge the assumption of inequalities as unidimensional but any type of conceptualization based on categorical thinking and it may be used to expose processes underlying social division and discrimination rather than differences between groups.

The inter-categorical approach contributes with a detailed mapping of health and disease across multidimensional population groups [6]. Furthermore, it contributes with an understanding of how the different dimensions interact in the production of health inequalities at intersecting social positions. It allows an examination of whether a health inequality is the result of a single dimension of inequality, the sum of two separate dimensions or the result of their interaction (i.e., exceeding the sum). In addition the approach shows the contingency and complexity of health inequalities [6].

### Underlying assumption 3: Categorization and labelling

In the government bill on alcohol, drugs, tobacco and gambling, categories are used in the representation of health inequalities. The underlying assumption of health inequalities as differences between population categories goes hand in hand with the quantitative epistemological assumption. Mohanty [64] argues that the analytical strategy in general, in which one population category is represented in contrast to an assumed (correct) norm, is a mechanism underlying marginalization. Thus, the discursive effect of the categorical logic is a way of thinking that does not only accentuate population categories as different from each other, but that also risk entrenching the stratification. This also has subjectification effects. For example, in the government bill, women drug users with experiences of violence are represented as a group with particular needs that the health care has difficulties attending to. This representation problematizes a certain group which are implicitly contrasted with an unproblematic norm.

Categorization does not only make subjects but also “places” such as “socioeconomically disadvantaged neighbourhoods”. The making of problematic subjects and places have lived effects in terms of experiences of marginalization. This works through a mechanism generating distance between people living in "disadvantaged areas," and “privileged areas,” or between the “vulnerable,” and “non vulnerable” [65, 66].

One dilemma is that categorization and norm-setting are useful power and governing technologies which enacts governable subjects, motivates resource distribution to particular groups [32, 33], and implicitly allows for collaboration between individuals and places with different problem representations [67]. In the government bill, this govern-mentality becomes particularly visible with respect to the preventive actions focusing on “risk groups,” and “disadvantaged neighborhoods,” which may risk marginalization and to fuel an “oppression Olympics”.

Categorisation, as the unidimensional representation of inequalities in health, risks reifying stereotypes and convoluting important knowledge. The labelling of the category “women” as “frail” is an example of such stereotyping found in the government bill, despite a higher prevalence of, for instance, gambling and mortality due to drug poisoning, among men than among women. Even though the labelling of this group as vulnerable may be grounded in an intention to “do good” it is likely to convolute the underlying structural forces, to disregard and undermine individual agency and is not likely to effectively improve the situation for women [9, 20, 29].

### Intersectional alternatives

The intra-categorical approach typically challenge categorization when it becomes disempowering but does not direct critique towards categorization itself. Considering for example some of the original texts on intersectionality, the emphasis of intersectional analysis is expressed as a kind of disentangling and recognition of different modes of oppression black women encounter [38, 60]. Thus, it may challenge the impression of homogenous population categories to some extent by highlighting important differences within categories but without completely disintegrating the concept of group belonging.

The anti-categorical approach on the other hand, put the challenging of categorization at the centre of analysis. As earlier mentioned, this means a rejection of using categories, even provisionally [6]. This stance is based in post-structural and postmodern ideas formulated around the performative power of policy and research (e.g., [68]). These suggest that the practice of categorization influences discourse and the discursive condition of social recognition in turn, forms the subject. Given the performative power of categorization in how it lays the ground for discrimination and social division it is a futile practice in a quest for equality. The contribution of the anti-categorical approach is not an alternative representation that can be readily adopted but rather the awareness and insight of the instability and arbitrariness of categorization and its potential implications.

The inter-categorical approach may partially challenge the idea of categorization by showing how inter-categorical intersectional inequalities in health vary with for example, time and space. Belonging to a population category may mean one thing for health in one setting but something else in another [6]. The approach does not direct critique to categorization itself but can, as an alternative to the categorization and labelling assumption, highlight their contingent significance for health inequalities.

## Discussion

### Summary of findings

We have critically analysed the problem representation of health inequalities in the proposed Swedish strategy on alcohol, drugs, tobacco and gambling as described in a government bill from 2021 [3]. This was done in order to examine its performative power on discourse, subjectification and lived effects, and to outline alternative intersectional representations.

Health inequalities were mostly described as a problem of differences in health between population groups in which one has worse health linked to alcohol, drugs, tobacco and gambling than the other. The recommended solutions raised in the government bill were: the importance of highlighting differences in health outcomes between population groups, and to consider how risk factors affect population groups differently. Hence, the problem representation reflected by these solutions is a lack of knowledge and use of knowledge. Furthermore, the particular type of knowledge discerned by this problem representation has a quantitative emphasis. Using an intersectional framework, we identified three underlying assumptions supporting the representation of health inequalities. The assumptions were related to quantification and objectivity, inequalities as unidimensional, and categorization and labelling. The analysis showed how the government bill, by opting into these partly overlapping assumptions, is part of, for instance, exercising epistemic violence and marginalization, and how it may focus more on describing differences than pointing towards solutions. The transformative part of the analysis then shows the potential of different intersectional approaches to challenge and disrupt these counteractive effects. These intersectional alternatives should not be interpreted as full-fledged solutions and we do not promote the use of one over the other. Rather, what our analysis shows is how these approaches can complement each other and that it is important to consider a multitude of approaches and knowledges to reduce marginalization and health inequalities.

### Practical implications and recommendations

The results direct attention to the performative power of problem representation in policy and its effects on discourses, subjects and living conditions. They imply that policy makers, researchers and other groups representing problems are made accountable for these effects. Therefore, we recommend such groups (ourselves included) to be cautious and critical of the problem representations they adopt. A systematic way of building such awareness in an organization or group could be the incorporation of time for reflection into an already existing working processes. The reflection could be further supported by a tool such as the questions of the WPR-approach or other questions that are directly related to the subject area.

The results also imply that deliberate consideration of alternative problem representations of health inequalities grounded in intersectionality could make public health policy more equality promoting by making difficult trade-offs explicit, which in turn may contribute to more transparent and democratic policy making (and knowledge making). Thus, another recommendation is to document the reflection we suggested making all the trade-offs identified explicit and transparent. This would improve the conditions for audit and evaluation and thus continuous learning and accountability.

### Reflections

The final step of the WPR-approach involves a critical analysis of one's own problematizations and assumptions. The choice of theoretical framework seems as a central starting point for such reflection. Intersectionality has been raised as a useful analytical tool for the analysis of health inequalities (e.g., [44]) but there are of course other frameworks that could have been used instead yielding different results. Choosing intersectionality meant some aspects of the problem representation went unexamined. For example, the policy also represented health inequalities as an untapped resource for economic growth. This problematization could have been examined with theories grounded in economics (e.g., [69]). Furthermore, using theories from governmentality studies could have explored what role the conceptualisation of risk had in the policy representation of health inequalities (e.g., [70]). Perhaps also an examination of the government bills’, factual and normative assumptions regarding as to whether rely on absolute (prevalence difference) or relative (ratio) measures of health inequalities would have identified other implications (e.g., [71]). The mix of legal (alcohol, tobacco, and gambling) and illegal (drugs including non-prescribed medical drugs and doping) substances and behaviors could also have been examined further. Even though concerted action on common risk factors has been pointed out as a strength there are also many differences in for example, judicial governance, norms and attitudes, and inequality patterns, and could therefore deserve a higher degree of differentiation in the strategy. Importantly, a more stringent use of the WPR-approach would perhaps have focused more on the specific solution to the problem of health inequalities, in this case gender and equity mainstreaming, and then worked backwards to elaborate on how this represents the problem as the failure to consider and integrate these perspectives during the implementation of the strategy.

Another issue with the choice of analytical framework is that intersectionality itself is a multifaceted concept that have been approached in many different ways, and the three approaches outlined by [6] are by no means exhaustive [41]. Even though the intention was to use different conceptualization of intersectionality in order to exhibit a multitude of interpretations and approaches some were excluded. For example, a conceptualization of intersectionality, which may be seen as transgressing the anti-categorical approach, view categories as continuously becoming through one another (e.g., [41, 72, 73]). This conceptualization is mostly grounded in Barads agential realism [74] and challenges both positivist and constructionist epistemology. It views scientific practice as a boundary making practice that makes particular configurations of reality knowable. Intersectionality grounded in this philosophy would provide an alternative representation of health inequalities that would be more contingent than that of the inter-categorical approach but without falling into complete relativism.

Finally, we reflect on our own assumptions and problem representation. In this study we have approached “the problem” of health inequalities from a policy perspective. The rationale we provide is the power of policy – the performative power beyond the direct impact of for example, specific regulations. By exposing certain epistemological assumptions underlying the policy representation of health inequalities, and by suggesting a diversification in epistemologies as a way forward, the issue of health inequalities is framed as a problem of knowing (or not knowing). An underlying assumption of this problem representation, which is also very similar to the problem representation found in the government bill, is that knowledge has the power to change practice. This assumption is central within the paradigm of evidence-based policy which has been criticized for constructing the value of “evidence” through the privileging and silencing of participants and discourses [15]. However, we do not elaborate on this particular assumption here, since it is not caught by our intersectional lens. It is an exclusion that we as scientists are accountable for [50] and that we take responsibility for by sharing in this final reflection.

## Conclusion

In this study we examined the representation of health inequalities in the Swedish policy on alcohol, drugs, tobacco and gambling. We conclude that the underlying assumptions: quantification and objectivity, inequalities as unidimensional, and categorization and labelling, makes and directs a discourse of health inequalities on specific subjects (e.g., vulnerable) with special needs (e.g., health care), in certain places (e.g., disadvantaged neighborhoods) and somewhat neglects the underlying processes of marginalization. Even though we raise some strengths with the problem representation it seems to be profoundly entangled with a system resisting the kind of change that the policy itself advocates for. We also conclude that intersectionality, if carefully used, seems to have a potential to enact empowered and complex subjects. However, tolerance and sensitivity in the dialogue across different knowledge traditions may be crucial for a successful transformation towards a truly equity-oriented public health policy and practice. 

## Data Availability

The material used in this study is publicly available at the website of the Swedish Government. URL: https://www.regeringen.se/pressmeddelanden/2021/03/fornyad-nationell-andts-strategi/
